# Recent Progress in Luminescent Cu(I) Halide Complexes: A Mini-Review

**DOI:** 10.3389/fchem.2021.816363

**Published:** 2022-01-25

**Authors:** Abraham Mensah, Juan-Juan Shao, Jian-Ling Ni, Guang-Jun Li, Fang-Ming Wang, Li-Zhuang Chen

**Affiliations:** School of Environmental and Chemical Engineering, Jiangsu University of Science and Technology, Zhenjiang, China

**Keywords:** copper(I) halides, luminescence, nitrogen ligands, sulfur ligands, phosphorus ligands

## Abstract

Copper(I) halide complexes are well sought-after materials due to their rich structural diversities and photophysical properties. Profoundly, there is a direct relationship between each structural variation and luminescence of these complexes, for a purported use. In this review, recent publications within the last 2 years about copper(I) halide complexes, centering on their structural dimensionalities with derivatives of nitrogen, phosphorus, and sulfur ligands, have been considered alongside their effects on luminescence.

## Introduction

The unprecedented reports about the usage of copper(I) halide complexes as a material in various fields are directly linked to their diverse structures, accompanied by unique properties. Precisely, for each diverse and distinct structure portrayed, there is a mechanism that controls the luminescence, which is not cryptic. Hence, acquiring an in-depth understanding of the structural variations of these materials vis-a-vis their emissions is essentially paramount to their application (Wang et al., 2019; Al-Masri and Almejled, 2020). In copper(I) halide complexes, mono-, di-, tri-, and tetra-coordinated complexes with substantive shapes like linear, trigonal, and tetrahedral have been reported. Higher coordinated states have also been recorded, but they mostly depend on the condition for the synthesis (Chen et al., 2003; Conry, 2005).

The atomic radius of the anion, the structural nature of the ligand, and the method for synthesis are part of the factors that control the coordination of copper(I) halide complexes. For instance, when the anionic size is relatively big as found in iodide and the extent of steric hindrance within the ligand is lower, multi-coordinated complexes are accessible contrary to mononuclear structures with properties differing from the above-stated points (Díez-González et al., 2010). On the other hand, the extent to which a ligand is fabricated may also determine the kind of mechanisms like ILCT, MLCT, charge-transfer-to solvent, LMCT, LLCT, inter-configurational CC, and *σ*-*a*_π_ transitions that can control the luminescence from copper(I) halide complexes (Kutal, 1990; Benito et al., 2016).

Although numerous reviews from different authors concerning the luminescence of Cu^I^, especially its complexes with halides, centering on the applications based on emission has been presented over the past years. For instance, in 2020, Ravaro et al. wrote about the “*luminescent copper(I) complexes as promising materials for the next generation of energy-saving OLED devices”* where they focused on OLEDs, the properties, and mechanism of Cu^I^ complexes as a good option for lighting types of equipment. Dinuclear N-heterocyclic carbene with Cu(I) complexes centered on the synthesis route and the structural properties have also been presented (Trose et al., 2018). Furthermore, Liu et al. (2017), Liu et al. (2018), and Hei et al. (2021) have also published many works about the above-mentioned complexes, under both reviews and research aspects mostly centering on their phosphorescence as an alternative source of light (Liu et al., 2017; Liu et al., 2018; Hei et al., 2021).

From our former research studies on inorganic–organic hybrid luminescent materials (Wang et al., 2016; Wang et al., 2018; Guo et al., 2019; Wang et al., 2021) and in order not to overlap, a mini-review on recently published works within the last 2 years on Cu(I) halide complexes and their structural relationship to luminescence is herein presented. Detail-wise, vivid descriptions about the various complexes, grounded on dimensionalities and the relationship between the established geometries and their impact on luminescence shall be hammered on. Nevertheless, it is imperative to note that not all publications concerning copper(I) halides may be captured herein. In the subsequent sections, structural variations of Cu(I) halide complexes based on various ligands and their respective dimensions vis-a-vis their photoluminescence shall be discussed.

## Nitrogen-Based Ligands

The combination of nitrogen-based ligands with CuI is the pioneering work that brought the synthesis and applications of copper(I) halide complexes to light (Hardt and Pierre, 1973). As per the propensity of nitrogen to donate electrons for coordination, they can be grouped into mono, di, tri, or multidentate forms depending on the number of atoms or hetero state by combining with other donating element(s) in a ligand (Bruns et al., 2010).

### Zero-Dimensional Nitrogen-Based Copper(I) Halide Complexes

**FIGURE 1:**
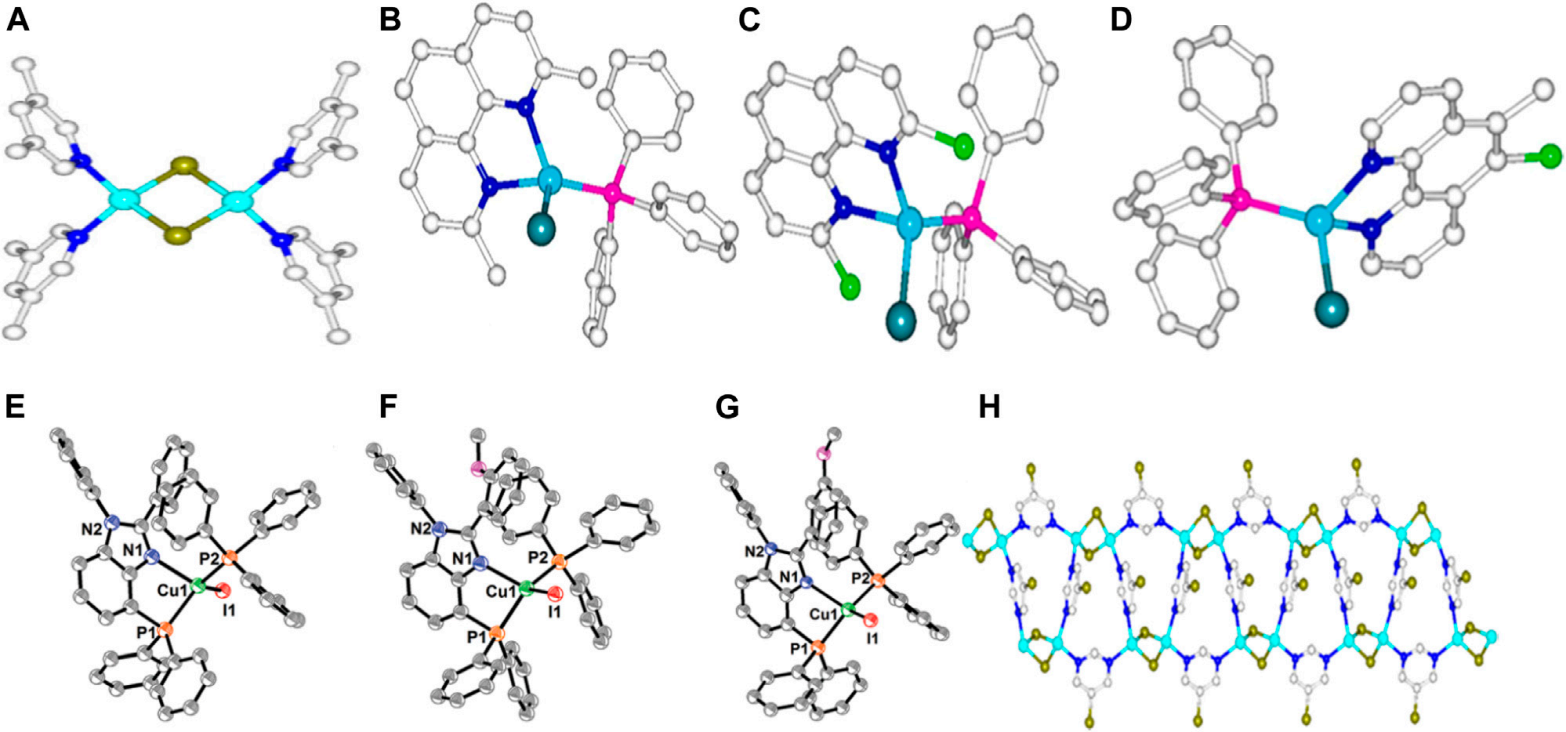
The crystal structures of complexes 1-8.

Based on both monoclinic and orthorhombic systems, *C2/c* and *Pbcn* space groups, two complexes, namely, Cu_2_Br_2_ (3,5-dimethyl-pyridine)_4_ and Cu_2_Br_2_ (5-bromo-pyrimidine) were formed from two functionalized pyrimidine ligands and cuprous bromide salt correspondingly. Only the 0D complex, **(1)** (Figure 1A) with the chemical formula Cu_2_Br_2_ (3,5-dimethyl-pyridine)_4_ shall be discussed here leaving the 1D structure (Cu_2_Br_2_ (5-bromo-pyrimidine) for the subsequent chapter. Complex **1** consists of a Cu_2_Br_2_ rhomboid dimer with two molecules of the ligand coordinating with each copper ion. At an excitation wavelength of 360 nm at room temperature, a green emission at 520 nm, controlled by MLCT was observed (Supplementary Figure S1). Due to the occupancy of CuBr and ligand subshells at valence and conduction bands correspondingly, **1** exhibited a bandgap of 1.536 eV with an IQY of 80.3% (Xu C. et al., 2020).

The combination of phenanthroline bearing different substituents, electron-withdrawing and donating groups at different positions with tpp and CuI yielded complexes **2–4** (Figures 1B–D) with the former ligand serving as a chelate agent. In general, the mononuclear copper metal coordinated with two nitrogen atoms within the phenanthroline, forming a five-sided figure with the phosphorus atom from the *tpp* at the apex. Furthermore, all the complexes had different space groups concurrently recorded as *C2/c*, *P-1*, and *P2*_*1*_ */n* for **2–4**. Even though the substituents could not have a lot of impact on their structural variations, however, they influenced the bandgap with the complexes bearing electron-donating groups displaying high values than those without. Besides, the MLCT process controlled the broad spectral emission in the solid-state at 605, **(A)** 620, and **(B)** 650 nm **(C)** in Supplementary Figure S2, respectively (Lv et al., 2021).

In a similar work with ^3^ (M + X) LCT-controlled emissions, the combination of another nitrogen- and phosphorus-based ligands, N^P and CuI, produced comparable five-sided Figures, **5,6** and **7** (Figures 1E–G) with a tunable temperature-dependent luminescence ranging from 589 to 568 nm (Supplementary Figures S3A–C) under the influence of a methoxy group. Although the complexes were collected on the same space group, nevertheless, there were variations in their internal bond length due to the positions of the methoxy substituent on the ligands. For instance, **7** with the smallest electron hindrance around the coordinating nitrogen atom had the longest Cu-N bond distance than **5** with a directly opposite property. Also, there were fewer intermolecular interactions among the ligands due to the methoxy substituents, which prevented the formation of dimensional structures higher than OD (Zhang et al., 2021).

### One-Dimensional Nitrogen-Based Copper(I) Halide Complexes

Complex **8** (Figure 1H) depicts the 1D structure synthesized using the same reagents as in **1** (Figure 1A) except that the substituents on the ligands are different. While 1 (Figure 1A) had bromide as a substituent, **8** (Figure 1H) contained two methyl groups at the third and fifth positions of the ligand connecting with the dinuclear copper metal to form a 1D chain, directly influencing its decomposition temperature. The transitions that mired the emission of 1 (Figure 1A) above were similar to that of 8 with a potential red color at 630 nm (Supplementary Figure S4) (Xu C. et al., 2020).

### Two-Dimensional Nitrogen-Based Copper(I) Halide Complexes

A series of multi-cluster copper(I) halide complexes, **9** and **10,** namely, MCC-1 and MCCH-2 had been created from the *TPPA* and CuI in MeCN/DMF by a solvothermal synthesis at 80 and 120°C, respectively. While the 2D structure **9**, a non-centrosymmetric complex, was made up of a neutral cluster of Cu_4_I_4_ and Cu_7_I_7,_ interconnecting to the ligand, **10**, a centrosymmetric complex, consisted of ionic clusters Cu_6_I_7_^−^ and Cu_6_I_5_^+^ interacting with six TPPA, closing the metal–metal bond distance. Aside from the similar broadband emissions exhibited by the complexes well-organized by the ^3^CC mechanism, the intensities of the luminescence from **9** increased while that of **10** decreased at the same conditions credited to the X/MLCT process (Supplementary Figure S5) (Yu et al., 2020).

## Phosphorus-Based Ligands

The ubiquitous applications of phosphine-based ligands, especially with Cu(I) halides in complex formation, are their knack to stabilize structures and the possession of varying coordination trends (Kaeser et al., 2013). The number of lone pairs of electrons for coordination in the derivatives of phosphorus-based ligands is dependent on the number of donating atoms it may contain (Aguirrechu-Comeron et al., 2018).

### Zero-Dimensional Phosphorus-Based Ligands Copper(I) Halide Complexes

Per the chemical structures of three nitrogen-based ligands, pyridine, quinoline, and 4-cynopyridine with phosphine (P1–P4, Scheme S1), five different CuI complexes **11–15**, (Supplementary Figure S6) were obtained in a mechano-chemical synthesis. Complex **11** with P1 and pyridine had the copper metal coordinating with the iodide, nitrogen, and phosphorus from the ligands forming a tetrahedral structure on a *P2*_*1*_ */c* space group with strong intermolecular interactions. Subsequently, complex **12**, collected on a triclinic system, and a *P-1* space group, containing P1 and quinoline, had a dinuclear coordination with the iodide, generating a planar structure. Surprisingly, **12** and **13** were obtained from the same reagents and procedure; however, they differed based on their Cu…Cu bond distance falling at 3.0566 Å and 2.8769 Å, correspondingly. Although **14** was made from P4 and pyridine, it had the same structural appearance as **12** except for its Cu…. Cu distance, which was short by 0.0516Å. The Cu…Cu bond distance in **15** with a cubane appearance was recorded at 2.77Å, being the shortest among all other complexes. The broad but varying solid-state emissions from the complexes were interdependent on the nature of the ligands with a corresponding mechanism such as ^3^(M + X)LCT and ^1^(M + X)LCT ((Supplementary Figure S6) (Egly et al., 2021).

A fused phosphorus-n combined with copper(I) halides in the CH_2_Cl_2_ solution yielded a set of dinuclear complexes with a general formula Cu_2_X_2_ (PՈN)_3_, (X = Cl Br and I). There were eight different complexes; however, only (Supplementary Figure S7) shall be centered on with their luminescence. Within the three structures, there were three substantive ligands, with two coordinating with different copper metals through their phosphorus atoms, while the third ligand joined the same metals by the nitrogen and the phosphorus groups completing a six-sided figure. Irrespective of their structural similarities, **16** was collected based on a monoclinic space group while 1**7** and **18,** being isostructural complexes, were crystallized on a triclinic system. Furthermore, their metal–metal bond distances differed slightly even among the isostructural complexes with **16** recorded at 2.883Å whereas **17** and **18** varied by 0.0274Å with the latter being the longest. All the complexes exhibited TADF and phosphorescence emissions generated from the ^3^M(XLCT) process, with 1**7** having the potential to serve as an OLED material due to its small activation energy gap at a wide temperature range (Supplementary Figure S7A) (Hofbeck et al., 2021).

### One-Dimensional Phosphorus-Based Copper(I) Halide Complexes

**FIGURE 2:**
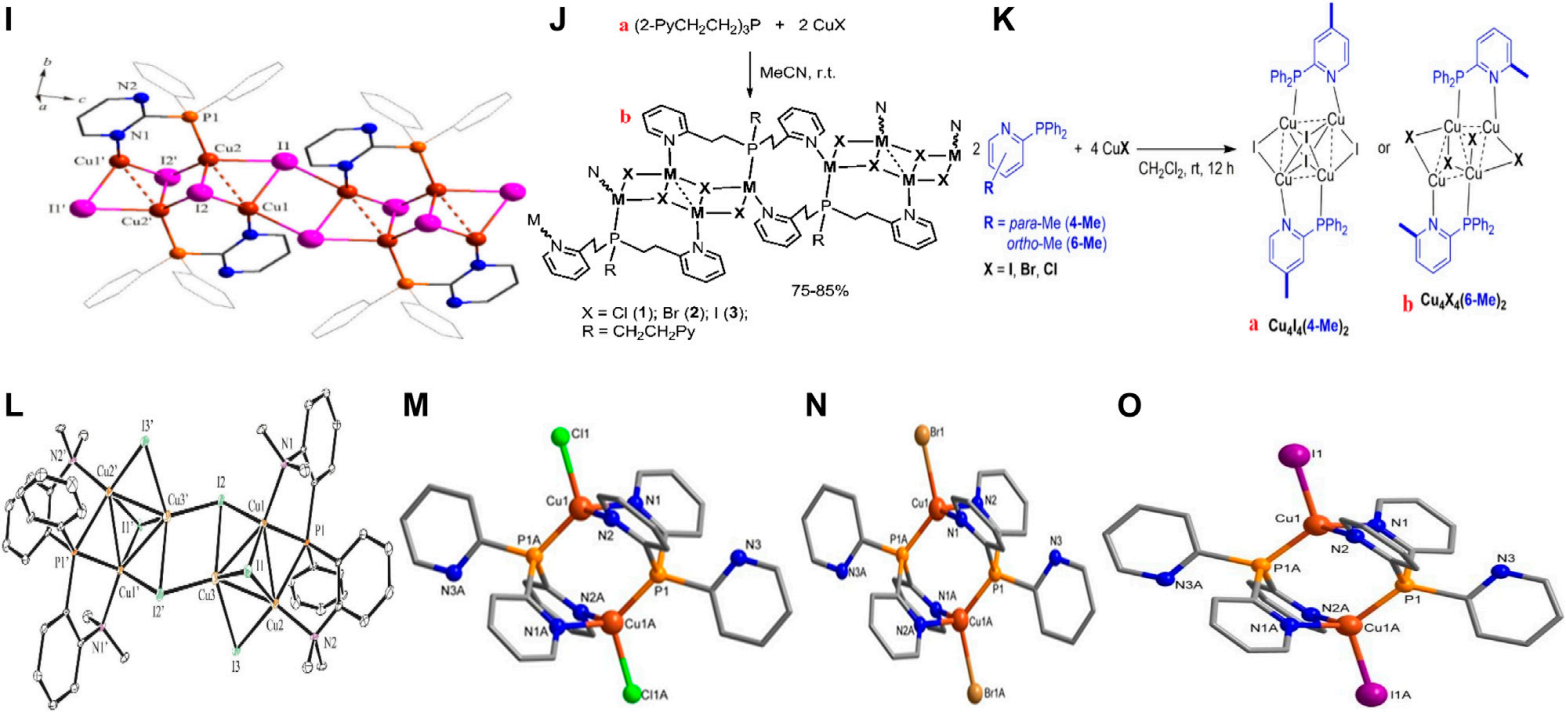
The crystal structures of complexes 19-25.

The 1D structure **19** (Figure 2I) resulted from the strong interaction between the polymer core and the bulky hemilabile ligand PymPPh_2_. From the molecular formula [Cu_2_I_2_PymPP_2_]n, the complex consists of a repetition of a double-stranded CuI inner core coordinating with the two different atoms within the tridentate ligand, forming a six-sided figure with the third donating atom left unattached. These extensions reduced the Cu…Cu distances paving way for cluster- centered and MLTC-induced emissions (Supplementary Figure S8A) (Davydova et al., 2020).

Artem’er and co-workers reported about three rare but similar 1D copper(I)-coordinating polymers with a Cu_4_I_4_ stair-step core and luminescence controlled by the nature of the halogen coordinating with the metal, using the synthesis pathway in **20a** (Figure 2J). A critical look at **20b** Figure 2J, an isostructural complex, to the remaining two had a copper from the inner core chains joined by a phosphorus atom at one end while a nitrogen group connected the other copper metal from another section of the Cu_4_I_4_ structure. The complexes gave broad emissions with clear separate colors through transition within ^3^ (M+X)LCT states (Supplementary Figure S9A) (Artem’ev et al., 2020).

Additionally, Boden et al. also fabricated a 2-(diphenylphosphino)pyridine ligand by placing a methyl group at either the *para* (4-Me) and *ortho* (6-Me) positions to the nitrogen atom to serve as a bridging ligand in Cu_4_X_4_ (X = I, Cl or Br) complexes. All the complexes, Cu_4_I_4_(4-Me)_2_, Cu_4_I_4_(6-Me)_2_, Cu_4_Br_4_(6-Me)_2,_ and Cu_4_Cl_4_(6-Me)_2_ structured in the form of either **21** a or b (Figure 2K), bearing similar crystal systems and spaces groups, had their CuX core between two ligands connected *via* the nitrogen and the phosphorus atoms. Notwithstanding, the structure of Cu_4_I_4_(6-Me)_2_ had a C_i_ symmetry due to the location of the complex in an inversion center while Cu_4_I_4_(4-Me)_2_ had its close to the C_i_ symmetry, bearing a Cu…Cu bond distance shorter than the former complex. Investigating the mechanism behind the thermochromic emission from the complexes (Supplementary Figure S10), the team concluded that the dual phosphorescence occurred due to the strong coordination pattern influencing ^3^M/XLCT and CC transitions (Boden et al., 2021).

### Two-Dimensional Phosphorus-Based Copper(I) Halide Complexes

Figure 2L consists of a complex **(22)** with a hexagonal CuI core obtained using a tridentate ligand consisting of phosphorus and nitrogen combined with copper(I) halides in MeCN, collected on a triclinic system with a *P2* space group. To the Cu_6_I_6_ framework, four of the copper metals were engulfed in a tetrahedral structure from connections between two iodides, nitrogen, and a phosphorus atom from the ligand. Due to the varying atoms, a strong intermolecular interaction made from CH…π and the phenyl groups collectively held the strong 2D structure. This rigid framework due to the diverse coordination within the complex enhanced its white-light emission in the solid-state at room temperature as well as the blue, white through to yellow-colored luminescence at 297 K, controlled by MLCT and XLCT mechanisms (Supplementary Figure S11A) (Xu K. et al., 2020).

### Three-Dimensional Phosphorus-Based Copper(I) Halide Complexes

Complexes **23–25** (Figures 2M, M and O) consist of three sets of 3D structures obtained through a mechanochemical synthesis using *py*_*3*_ *p* and CuX salts (X = Cl, Br, and I). The three complexes were isostructural in which the copper metal had distorted the tetrahedral shape by coordinating with two nitrogen atoms, phosphorus, and the respective halides. Albeit the weak intermolecular interactions within the complexes, they were undoubtedly the holding force within the 3D framework around an extended Cu…Cu bond distances. Two luminescence phenomena, TADF and phosphorescence correspondingly from ^1^M+X(LCT) and ^3^M+X(LCT) excited states displayed by the complexes were reconfirmed using DFT and TDFT approaches. (Baranov et al., 2020).

Two varying CuI complexes Cu_3_I_3_S and Cu_4_I_4_ denoted as **26** and **27** in Supplementary Figure S13 were joined together by a multidentate ligand *TPSA* to form a 3D structure **28** (Supplementary Figure S13), held in shape by strong hydrogen bonds. The linkage between the adjusted coordination conforms from the ligand, the Cu_3_I_3_S cluster, and the Cu_4_I_4_ core with a cubane-like ring resulted in a diverse rigid structure. Again, the proximity of metals to each other in the Cu_3_I_3_Se cluster resulted in a strong cuprous interaction than observed in the Cu_4_I_4_ ring. Hence, in the solid-state and at room temperature, the complex gave a red emission as a result of the metal–metal interaction in the Cu_3_I_3_S and XLCT from the Cu_4_I_4_ (Supplementary Figure S13A) (Zhao et al., 2021).

## Sulfur-Based Ligands

Aside from nitrogen and phosphorus, the ligands that dominate in terms of coordination with copper(I) halides are the sulfur-based type owing to their ability to mix with other ligands serving as either an auxiliary, with the halogen as a bridge (Supplementary Figure 14A), or as a link joining the CuX (X = halogen) core (Supplementary Figure 14B). On the other hand, the copper(I) in the midst of derivatives of sulfur ligands may also serve as a bridge to a halide to create diverse structures (Supplementary Figure 14C) (Tsuge et al., 2016).

### One-Dimensional Sulfur-Based Copper(I) Halide Complexes

Complex **29** in Supplementary Figure S15 consists *H6ma* and *H*_*2*_ *dtdn*, two mecaptonicotinic ligands and copper(I) halide in a 1D **(29)** and two 2D copper(I) halide–coordinating polymers (Supplementary Figure S16) from Scheme S2. The disorderliness within the ligand was the main reason for the extended Cu…Cu bond length, denying a CC-based emission but rather XMCT-assisted luminescence at 620 nm in Supplementary Figure S15A (Hassanein et al., 2020).

### Two-Dimensional Sulfur-Based Complexes With Copper(I) Halides

The 2D structures obtained through Scheme S2 above are displayed in Supplementary Figure S16 below as **30** and **31** with their respective emission spectra, **(A,B)** Supplementary Figure S16A,B. At harsh conditions, both ligands (*H6ma* and *H*_*2*_ *dtdn*) with the metal salt yielded complex **30** crystallizing on a monoclinic system with a P2_1_/c space group without a halogen anion in the structure. However, when the conditions were changed, the same reagents produced **31,** indicating the presence of the halide in the structure through the P2_1_/c space group. The exclusion of the halide ion from complex **30** could not account for a halogen-assisted emission but rather a direct MLCT, with a reverse mechanism in **31**, whereas the 2D structure of **30** was generated from the coordination between the zig-zag–shaped sulfur atoms and the copper metal; a 1D core of Cu_2_I_2_ linkage with the ligands produced the 2D appearance of **31** (Hassanein et al., 2020).

Dimensional coordinating polymers, with 2D (**32** in Supplementary Figure S17) and three 3D structures (Supplementary Figure S18), were presented by Liang et al. with structural variations emerging from either the type of the halide or the metal-to-ligand ratio. The complexes were made from *hpzt* and CuX (X = I, Br, and Cl) with a 1:3 ratio of the ligand to CuI yielding 32 consisting of two protonated ligands, connected by Cu^+^ and I^−^ in a distorted tetrahedral structure. The 2D structure was generated out of the interaction between Cu_6_S_6_ and Cu_4_S_3_I units, creating short Cu…Cu bond distances, capable of influencing CC-assisted emissions; however, the complex was non-emissive probably due to how the molecules arranged themselves in the stacking modules (Liang et al., 2020).

### Three-Dimensional Sulfur-Based Copper(I) Halide Complexes

Complexes **33–35** (Supplementary Figure S18) are the 3D structures obtained from the same synthesis procedure used for the synthesis of **32**. From the structures, **33** crystallized on a monoclinic system and *I2/m* space group with the 3D figure created around two main interaction building blocks; 1) CuN_2_SCl and Cu_4_S_4_ with a tetrahedral outlook and 2) hexagonal Cu_6_S_6_ structures, which only showed temperature-regulated emission through ^3^CC (Supplementary Figure S18). The next crystal **34**, with a monoclinic but *P2/n* space group, had its 3D outlook made *via* the connections between a copper metal with a tetrahedral shape formed from the coordination between two ligands and three chloride ions, with Cu_4_S_4_ and a chloride ion serving as a bridge. On the other hand, **35**, an isostructural to [Cu_4_(pzt)_3_ I]n, had an interaction between Cu_6_S_6_ and Cu_2_Br_2_ forming the 3D network (Liang et al., 2020).

## Conclusion

In conclusion, when copper(I) halide complex material is structurally stable, the probability of exhibiting combined properties such as solubility in solutions, thermal permanency, and efficient luminescence is highly attainable for its usage. Dimensional complexes from copper(I) halide are mostly stable due to the extended bonds created among the coordinating atoms, which may enhance their emissions. Among other conditions, two factors that may play a role during the build-up to the dimensional copper(I) halide complex are hydrogen bonds and intra-ligand interactions. Comparatively, the higher the dimensionality, the more stable the structure may be due to numerous patterns of interconnecting bonds. Although most dimensionless copper(I) halides may show emissions through MLCT, CC, LMCT, LL, and XLCT, mechanisms are just like the dimensional type; however, their differences in terms of application lie within their stabilities.
