# Supplementary material for: Recent Progress in Luminescent Cu(I) Halide Complexes: A Mini-Review
Source: Front Chem. 2022 Jan 25;9:816363. doi: 10.3389/fchem.2021.816363 (PMC8822502; doi:10.3389/fchem.2021.816363)
Supplement: Supplementary file 1 [file DataSheet1.docx]

**Supplementary Material**


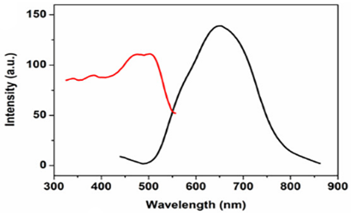


A

Figure S 1The PL spectra (A) of complex 1(Xu et al., 2020a)


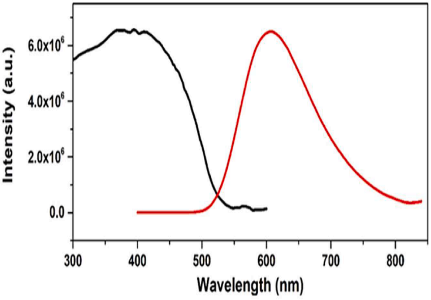

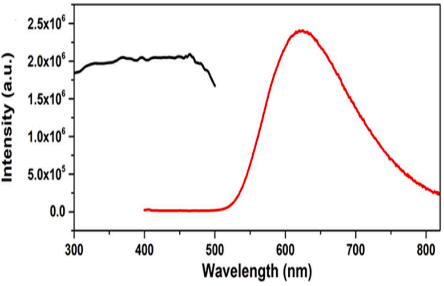

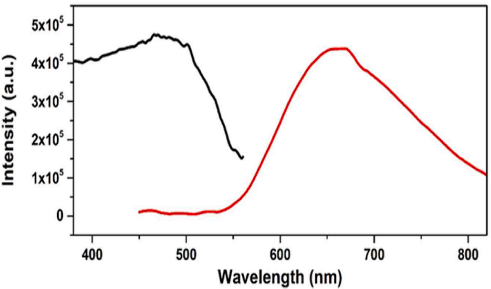


A B C

Figure S 2 The PL spectra (A-C) complexes 2-4 (Lv et al., 2021)


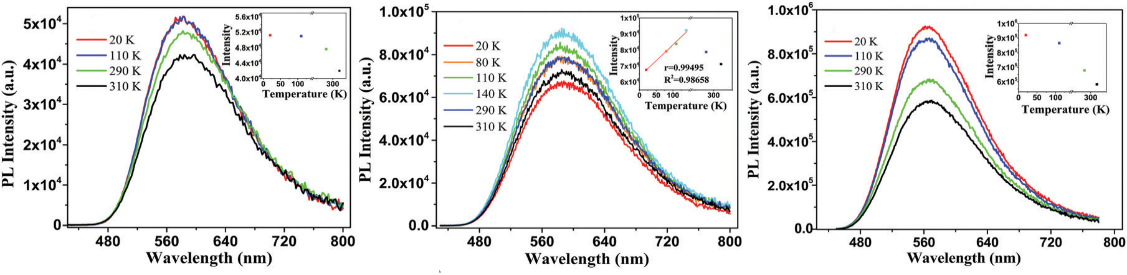


A B C

Figure S3 The PL spectra (A-C) complexes 5-7 (Zhang et al., 2021)


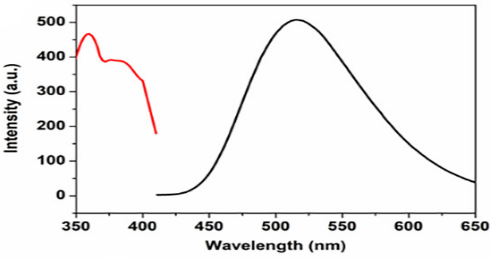


8 A

Figure S4 PL spectrum (A) of complex 8(Xu et al., 2020b)


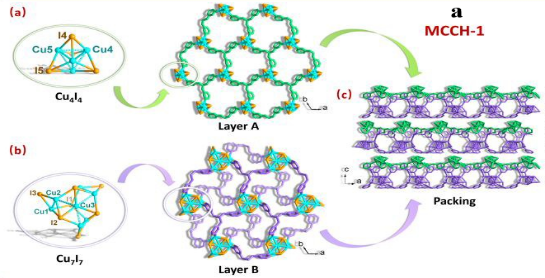

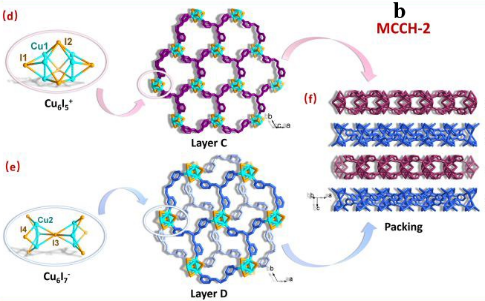

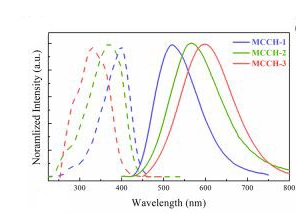


9 10 A

Figure S5 The formation of complexes 9 and 10 and their PL spectra (A)(Yu et al., 2020)


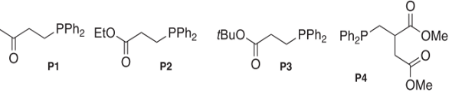


Scheme S1 Phosphine ligands P1-P4(Egly et al., 2021)


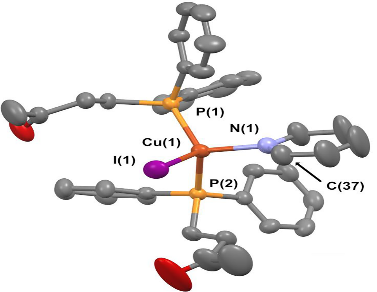

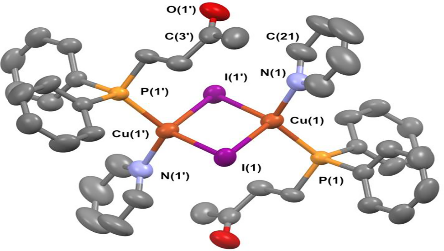

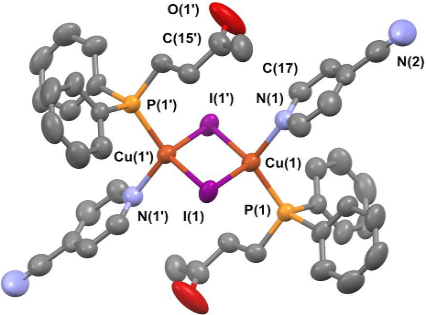


11 12 13


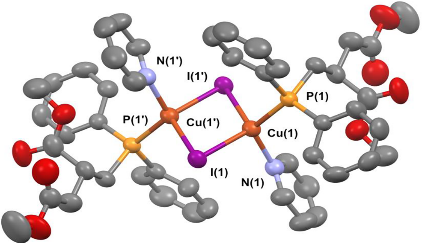

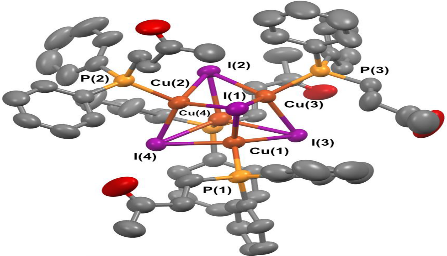

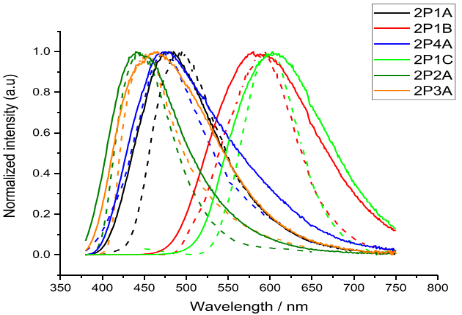


14 15 A

Figure S 6 Structures of complexes 11-15 and their PL spectra (A)(Egly et al., 2021)


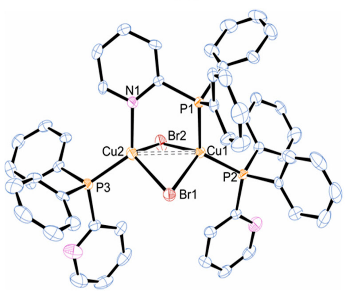

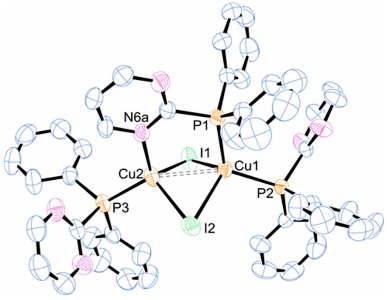

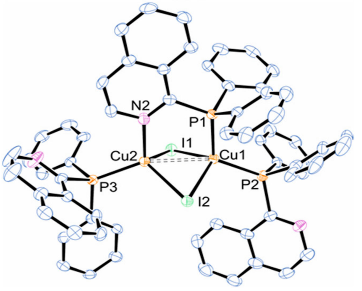


16 17 18


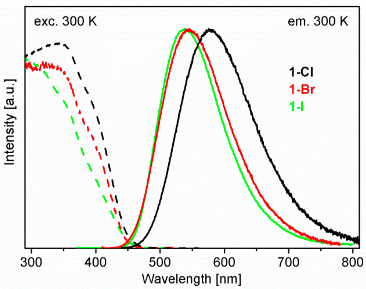


A

Figure S7 Structures of complexes 16-18 and their PL spectra (A)(Hofbeck et al., 2021)


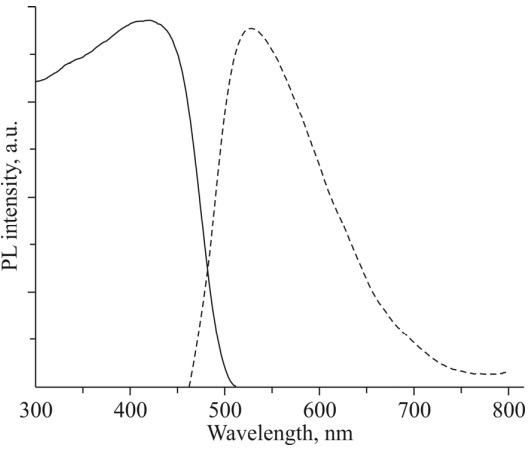


19 A

Figure S8 The PL spectra (A) of complex 19 (Davydova et al., 2020)


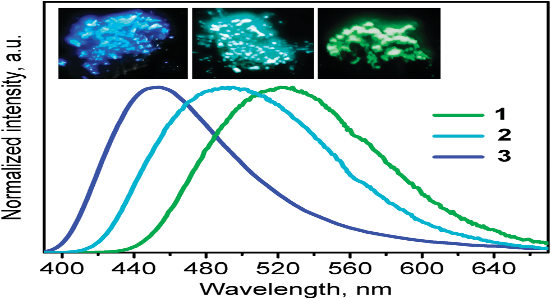


A

Figure S9 The solid emission spectra of complex 20 at 300 K (A)(Artem’ev et al., 2020)


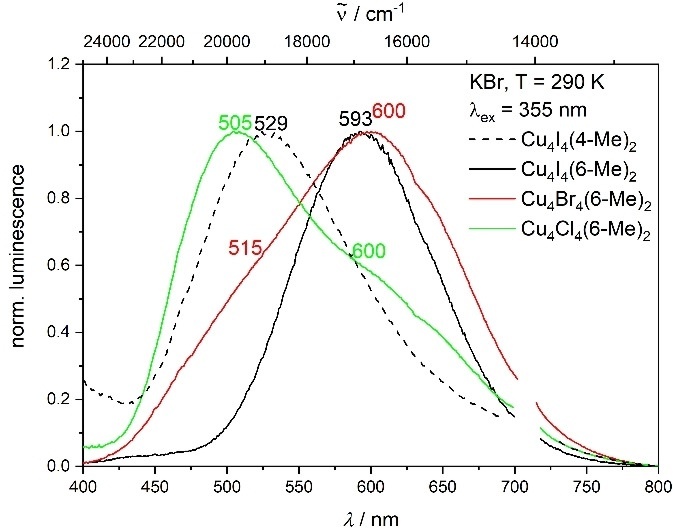


A

Figure S10 The solid emission spectra of complex 21 at 290 K (A)(Boden et al., 2021)


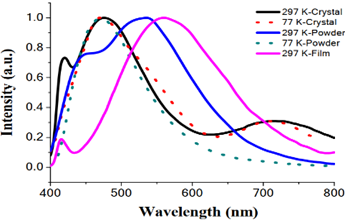


A

Figure S11 The emission spectra of complex 22 at different temperatures (A)(Xu et al., 2020c)


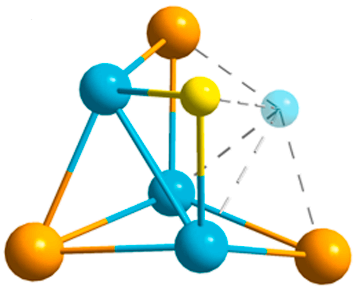

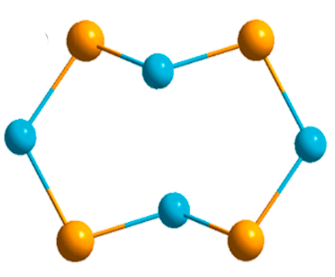

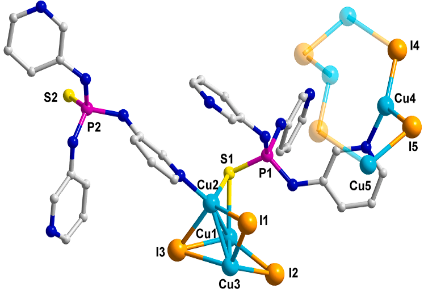

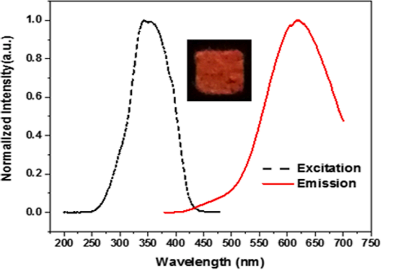


26 27 28 A

Figure S13 Formation of complex 28 from 26 and 27 and the PL spectra (a)(Zhao et al., 2021)


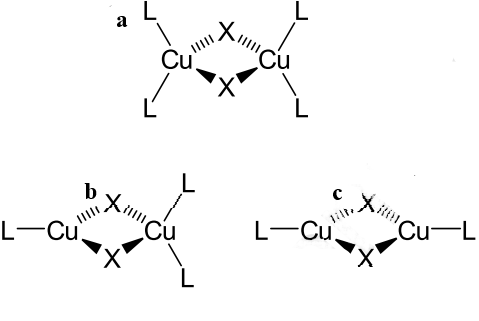


Figure S14 Common coordination patterns of sulfur ligands(Tsuge et al., 2016)


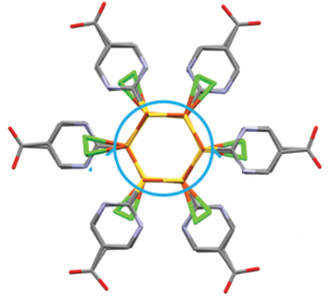

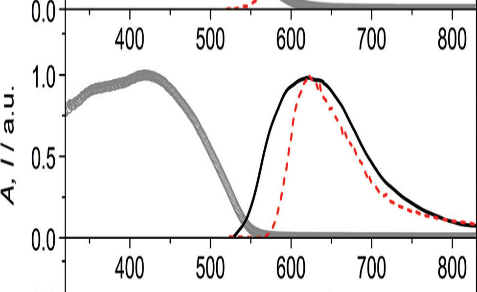


29 A

Figure S15 Crystal structure of complex of 29 and the PL spectra (A)(Hassanein et al., 2020)


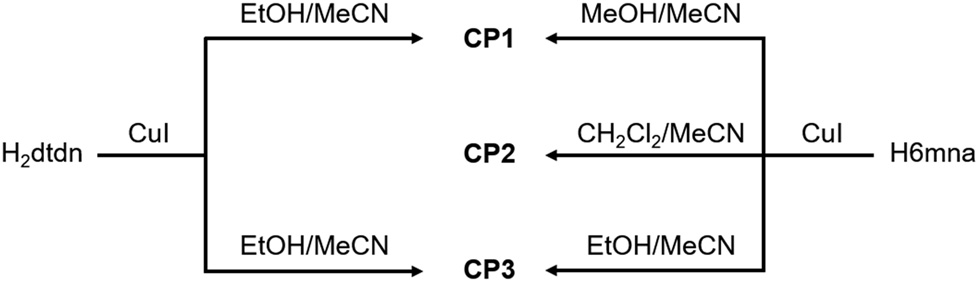


Scheme S2 Synthetic routes for of complex 29(Hassanein et al., 2020)


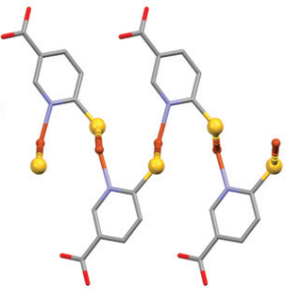

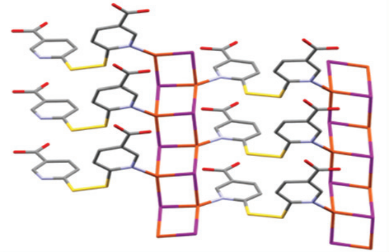

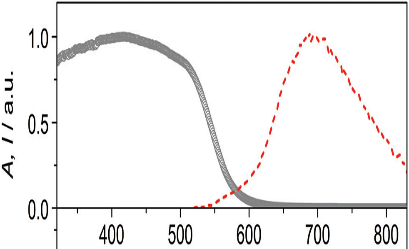

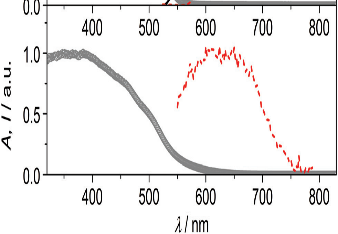


30 31 A B

Figure S16 Crystal structures of complexes 30 and 31 with the respective PL spectra (A and B)(Hassanein et al., 2020)


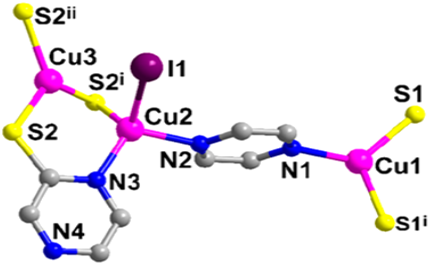


Figure S17 Crystal structure of complex 32


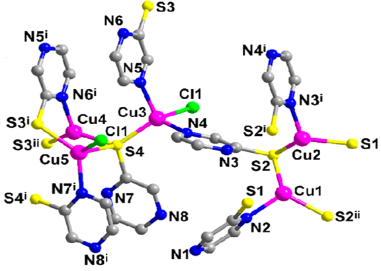

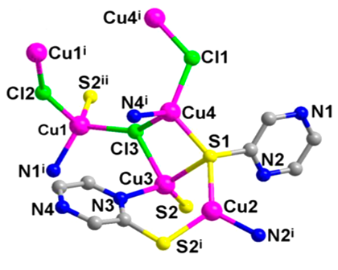

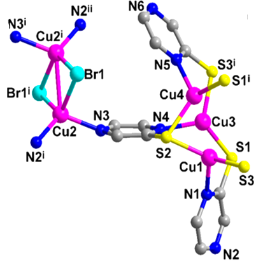

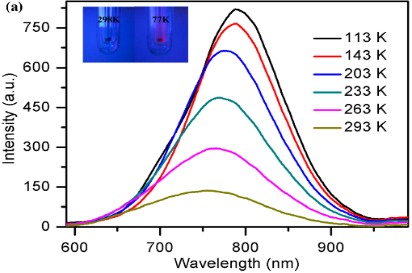


33 34 35

Figure S18 Crystal structures of 38-40 and the PL spectrum of 38 (A)

REFERENCE

Artem’ev, A.V., Baranov, A.Y., Rakhmanova, M.I., Malysheva, S.F., and Samsonenko, D.G. (2020). Copper(i) halide polymers derived from tris[2-(pyridin-2-yl)ethyl]phosphine: halogen-tunable colorful luminescence spanning from deep blue to green. *New Journal of Chemistry* 44(17)**,** 6916-6922. doi: 10.1039/D0NJ00894J.

Boden, P., Di Martino-Fumo, P., Busch, J.M., Rehak, F.R., Steiger, S., Fuhr, O., et al. (2021). Investigation of Luminescent Triplet States in Tetranuclear Cu-I Complexes: Thermochromism and Structural Characterization. *Chem European J* 27(17)**,** 5439-5452. doi: 10.1002/chem.202004539.

Davydova, M.P., Rakhmanova, M.I., Bagryanskaya, I.Y., Brylev, K.A., and Artem'ev, A.V. (2020). A 1D Coordination Polymer Based on CuI and 2-(Diphenylphosphino)Pyrimidine: Synthesis, Structure and Luminescent Properties. *J. Struc Chem* 61(6)**,** 894-898. doi: 10.1134/S0022476620060086.

Egly, J., Bissessar, D., Achard, T., Heinrich, B., Steffanut, P., Mauro, M., et al. (2021). Copper(I) complexes with remotely functionalized phosphine ligands: Synthesis, structural variety, photophysics and effect onto the optical properties. *Inorg Chimi Acta* 514. doi: 10.1016/j.ica.2020.119971.

Hassanein, K., Cappuccino, C., Amo-Ochoa, P., Lopez-Molina, J., Maini, L., Bandini, E., et al. (2020). Multifunctional coordination polymers based on copper(i) and mercaptonicotinic ligands: synthesis, and structural, optical and electrical characterization. *Dalton Trans* 49(30)**,** 10545-10553. doi: 10.1039/d0dt01127d.

Hofbeck, T., Niehaus, T.A., Fleck, M., Monkowius, U., and Yersin, H. (2021). P boolean AND N Bridged Cu(I) Dimers Featuring Both TADF and Phosphorescence. From Overview towards Detailed Case Study of the Excited Singlet and Triplet States. *Molecules* 26(11). doi: ARTN 341510.3390/molecules26113415.

Lv, L., Wang, S.Q., and Liu, W. (2021). Copper iodide organic-inorganic hybrid chelating clusters as luminescent coating materials. *Inorg Chimica Acta* 518. doi: ARTN 12024110.1016/j.ica.2020.120241.

Tsuge, K., Chishina, Y., Hashiguchi, H., Sasaki, Y., Kato, M., Ishizaka, S., et al. (2016). Luminescent copper(I) complexes with halogenido-bridged dimeric core. *Coord Chem Rev* 306**,** 636-651. doi: 10.1016/j.ccr.2015.03.022.

Xu, C., Li, Y., Lv, L., Lin, F., Lin, F., Zhang, Z., et al. (2020a). Synthesis, characterization, luminescence properties of copper(I) bromide based coordination compounds. *Inorganica Chimica Acta* 512**,** 119893. doi: <https://doi.org/10.1016/j.ica.2020.119893>.

Xu, C., Li, Y., Lv, L., Lin, F., Lin, F., Zhang, Z., et al. (2020b). Synthesis, characterization, luminescence properties of copper(I) bromide based coordination compounds. *Inorg Chim Acta* 512**,** 119893. doi: 10.1016/j.ica.2020.119893.

Xu, K., Chen, B.L., Zhang, R., Liu, L., Zhong, X.X., Wang, L., et al. (2020c). From a blue to white to yellow emitter: a hexanuclear copper iodide nanocluster. *Dalton Trans* 49(18)**,** 5859-5868. doi: 10.1039/c9dt04701h.

Yu, M., Liu, C., Li, S., Zhao, Y., Lv, J., Zhuo, Z., et al. (2020). Constructing multi-cluster copper(i) halides using conformationally flexible ligands. *Chem Commun (Camb)* 56(53)**,** 7233-7236. doi: 10.1039/d0cc02472d.

Zhang, B., Zhang, J., Sun, A.H., Liu, C.M., Gu, M.S., Chen, Y.H., et al. (2021). Efficiently luminescent mononuclear copper iodide complexes with sterically hindered iminephosphine chelating ligands. *New J of Chem* 45(19)**,** 8763-8768. doi: 10.1039/d1nj00622c.

Zhao, Y.F., Yu, M.X., Jiang, F.L., Chen, L., and Hong, M.C. (2021). A red-emissive 3D framework with the coexistence of copper-iodide clusters and rings as a luminescent ratiometric thermometer. *Inorg Chem Commun* 127. doi: ARTN 10851710.1016/j.inoche.2021.108517.
